# Epidemiological Trends of Coronavirus Disease 2019 in Sierra Leone From March 2020 to October 2021

**DOI:** 10.3389/fpubh.2022.949425

**Published:** 2022-06-29

**Authors:** Zhiguo Liu, Liping Gao, Chuizhao Xue, Chunchun Zhao, Tiezhu Liu, Alie Tia, Lili Wang, Junling Sun, Zhenjun Li, Doris Harding

**Affiliations:** ^1^National Institute for Communicable Disease Control and Prevention, Chinese Center for Disease Control and Prevention, Beijing, China; ^2^Sierra Leone-China Friendship Biological Safety Laboratory, Freetown, Sierra Leone; ^3^National Institute for Viral Disease Control and Prevention, Chinese Center for Disease Control and Prevention, Beijing, China; ^4^National Institute of Parasitic Diseases, Chinese Center for Disease Control and Prevention (Chinese Center for Tropical Diseases Research), NHC Key Laboratory of Parasite and Vector Biology, WHO Collaborating Centre for Tropical Diseases, National Center for International Research on Tropical Diseases, Shanghai, China; ^5^Key Laboratory of Surveillance and Early-Warning on Infectious Disease, Chinese Center for Disease Control and Prevention, Beijing, China; ^6^Central Public Health Reference Laboratories, Ministry of Health and Sanitation, Freetown, Sierra Leone

**Keywords:** epidemiological trends, COVID-19, positivity rate, three waves, geographic distribution

## Abstract

Coronavirus disease 2019 (COVID-19), a serious public health challenge the world over, has led to significant health concerns in Sierra Leone. In the present study, epidemic indices, such as the number of cases, positivity rate, reproduction rate (R0), case fatality rate (CFR), age, and sex, were used to characterize the epidemiological trends of COVID-19. As of October 31, 2021, a total of 6,398 cases and 121 related deaths had been confirmed. The total number of COVID-19 reverse transcription polymerase chain reaction (RT-PCR) tests conducted to October 31, 2021, was 249,534, and the average positivity rate was 2.56%. Three waves of COVID-19 were recorded, occurring during weeks 15–46 in 2020 (2,369 cases), week 47 in 2020 to week 16 in 2021 (1,665 cases), and weeks 17–43 in 2021 (2,364 cases), respectively. Remarkably, there was no increase in the numbers of confirmed COVID-19 cases despite rising test numbers throughout the three waves. Moreover, three high R0 values were observed before each wave. The number of positive cases significantly correlated with positive numbers of international arrivals (*P* < 0.01), deaths (*P* < 0.01), and the positivity rate of tested samples (*P* < 0.01). Moreover, all of the deaths occurred during the peak of the three waves. Our results indicate that there was a low level of COVID-19 epidemic in Sierra Leone and that COVID-19's introduction led to local transmission. It is vital to fight against the spread of SARS-CoV-2 from the source of origin by strengthening testing and management of people entering the country. Our findings will provide important clues for expanding sample screening and will contribute to the reasonable allocation of medical resources.

## Background

The coronavirus disease 2019 (COVID-19) pandemic has been one of the largest outbreaks of a viral infectious disease in recent decades. The globalization of COVID-19 resulting from the evolution of a local epidemic in China to a worldwide pandemic has resulted in significant risks to population health, especially in some undeveloped countries (1, 2). Moreover, in undeveloped countries with fragile health systems and overcrowded living conditions, implementing preventive measures such as physical distancing and lockdowns is difficult, as a result of which loss of income, increased food prices, and reduced access to non–COVID-19 health services can have dire consequences both in the short and in the long term (3).

Located on the west coast of Africa, Sierra Leone, being one of the most undeveloped countries in the world, has a particularly vulnerable public health system (4, 5). Even though Sierra Leone reacted quickly to the threat of COVID-19, implementing policies to contain the spread of severe acute respiratory syndrome coronavirus 2 (SARS-CoV-2) (6), COVID-19 emerged as a serious public health challenge due to the weak health system, poverty, cultural norms, and limited medical resources (7, 8) [e.g., a single ventilator was available for the whole population of Sierra Leone in the initial stage of the COVID-19 pandemic (1)], which led to significant health concerns and socioeconomic issues. A related study showed that people lost their jobs and had difficulty providing food for their families as a consequence of COVID-19 lockdowns (1). Although health care workers in Sierra Leone showed good knowledge and positive attitudes and practices regarding COVID-19, they maintained the view that their health care facilities were ill-prepared to respond adequately to the SARS-CoV-2 outbreak (9). Furthermore, another study has suggested that the COVID-19 pandemic adversely affected tuberculosis care delivery in Sierra Leone (10). Additionally, a significant reduction in malaria diagnosis in those <5 years of age was noted in April 2020 compared to April 2019 due to the COVID-19 lockdowns (11).

However, characterization of the epidemiological features of COVID-19 is crucial for the development and implementation of effective control strategies to reduce the socioeconomic effects of the COVID-19 pandemic. Here, we report the results of a descriptive, exploratory analysis of all of the cases diagnosed between March 2020 to October 2021 in Sierra Leone to better understand the epidemic's progression and to formulate targeted strategies to contain current and future viral outbreaks.

## Methods and Materials

### Ethics Statement

This study was supported by the third phase of technical assistance project for the fixed biosafety laboratory in Sierra Leone from China CDC and was approved by the Commission of Ethics and Science Censor of the Sierra Leone Ministry of Health and Sanitation. Our survey adhered to the medical ethics of domestic laws and regulations. Furthermore, in order to fully respect and protect the privacy of enrolled patients, personal data were not collected by the survey.

### Data Source and Data Process

A total of 6,398 positive cases were detected in Sierra Leone between March 2020 and October 2021; of these, 1,647 positive cases were detected by the China–Sierra Leone Biosafety Laboratory (Jui P3 lab), and the other 4,751 positive cases were identified from the COVID-19 Situational Report issued daily by the Sierra Leone Ministry of Health and Sanitation. After being stripped of personal identifying details, all of the data were processed and cross-checked by two trained qualified health workers. Moreover, the “wave” was defined according to the average threshold (criterion 3: the average week between the end of the first wave and the start of the second wave) (12), while weekly confirmed cases in 30 as a threshold value. The “reproduction rate” [brief definition: reproduction number (R0) is the number of secondary cases which one case would produce in a completely susceptible population (13)] and “stringency index” [brief definition: COVID-19: the stringency index is a composite measure based on nine response indicators including school closures, workplace closures, and travel bans, rescaled to a value from 0 to 100 (100 = strictest)] (14) data associated with COVID-19 in Sierra Leone were extracted from the public website “Our World in Data” (https://ourworldindata.org/covid-cases). Moreover, the average values of the reproduction rate and stringency index for every seven days were calculated and used for epidemics trend analysis. The epidemiological relationship between positive cases among incoming travelers and another four basic epidemiological indices (number of positive cases, health worker cases, death numbers, and positivity rate of tested samples) was determined by the R software (R Foundation for Statistical Computing, Vienna, Austria). A *P* value <0.01 was statistically significant.

### Strategy for Testing Infection Samples

Nasal and/or pharyngeal swab samples collected from each suspected COVID-19 case were sent to the laboratory for confirmation via reverse transcription polymerase chain reaction (RT-PCR). RNA extraction of the samples (P3 lab) and RT-PCR amplification (P2 lab) used the Shuoshi Biotechnology (Jiangsu, China) 2019-nCOV detection kit. The RT-PCR assays targeting the open reading frame 1ab (*ORF1ab*) and nucleocapsid (*N*) genes of SARS-CoV-2. The procedures of the experiment were performed, and the results were interpreted according to the manufacturer's instructions. Briefly, the cycle threshold value was ≤ 37, and positive results required both *ORF1ab* and *N* gene results to be positive or for any single target positive repeat test to remain positive. Briefly, a confirmed case corresponds to a person who has a positive result of SARS-CoV-2 from a RT-PCR test, tested specimen including throat swab, nose swab, or saliva et al. (12, 15).

## Results

### Epidemiological Trends According to Weeks of the COVID-19 Pandemic

**Figure 1:**
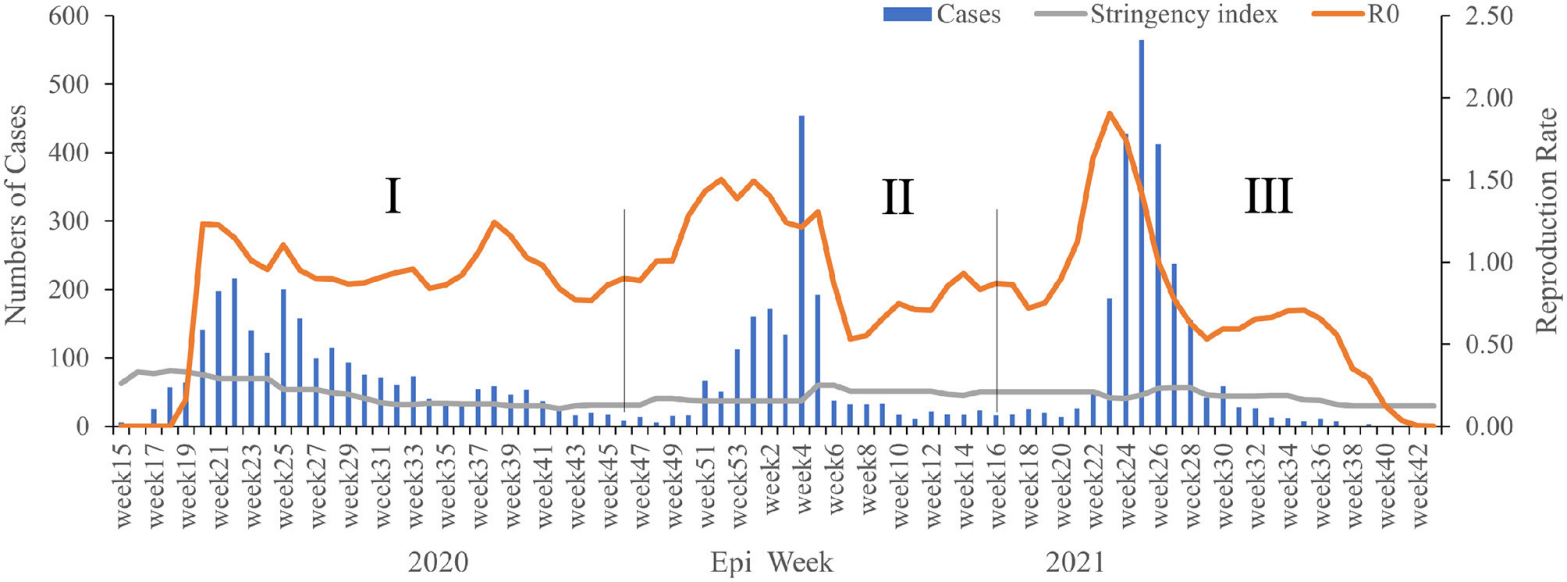
The weekly epidemiological trend of COVID-19 in Sierra Leone from March 2020 to October 2021. The vertical lines identify the thresholds for the wave's duration.

Based on the profile of weekly reported cases, three waves of significant COVID-19 cases were recorded during the study period. The first wave occurred from weeks 15–46 in 2020, peak in weeks 17–41, where a total of 2,771 (>35%) confirmed cases were found (Figure 1). The wave involved ongoing spread of SARS-CoV-2 for up to seven months (March–November 2020), and the greatest number of cases (216) was identified in week 22 in the first wave. The second wave started in week 47 in 2020 (November) and end in week 16 (April) in 2021, but cases declined suddenly in week 5 in 2021 (January). The spread of SARS-CoV-2 continued at a lower level for a period of time after that, until week 21, when confirmed cases began increasing again gradually (Figure 1). Then, the third wave was recorded from week 17 (April) until week 43 (October) in 2021, the epidemic peak in week 22–32 (June-August), when a total of 2,195 positive cases were detected, and the average weekly reported number of cases was 199.5. The highest epidemic week was in week 25 in 2021, with 565 confirmed cases, then in week 24 in 2021 (*n* = 428) and week 26 in 2021 (*n* = 412) (Figure 1). After these weeks, fewer confirmed cases were identified, with almost no positive cases diagnosed in weeks 40–44.

### Epidemiological Relationship Between the Reproduction Rate, Stringency Index, and Three Waves

There was an obvious epidemiological relationship between confirmed case numbers, the reproduction rate (R0), and the stringency index in our study. Remarkably, the highest R0 value was recorded just before every wave (Figure 1), although there were also three peak R0 values among the three waves, including 1.23 in week 20 in 2020, 1.5 in week 52 in 2020, and 1.91 in week 23 in 2021 (Figure 1). Moreover, R0 fluctuated continuously in a relatively stable range (0.53–1.91) among the three waves, but there was a significant decline in the R0 after the third wave. Similarly, the stringency index showed an epidemiological relationship with the reproduction rate and three waves. During the first wave, the stringency index decreased continuously, but it increased weakly thereafter in the late (mid-) stage of the second and third waves (Figure 1). Generally, the stringency index decreased gradually from the first wave (80.95 in week 18 in 2020) to the third wave (29.63 in week 43 in 2021).

### Positivity Rate and Number of Deaths Among Tested Samples

**Figure 2:**
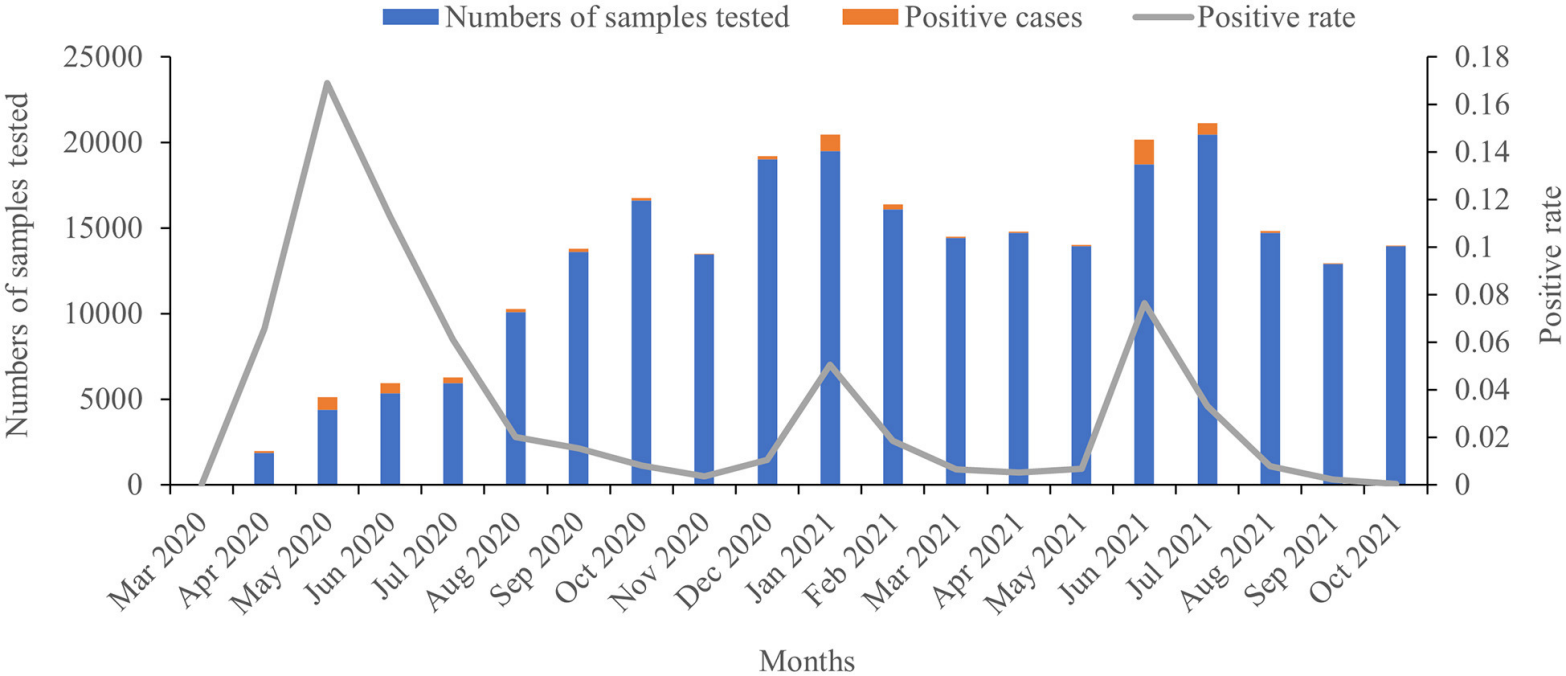
Numbers of tested COVID-19 samples and the positivity rate.

**Figure 3:**
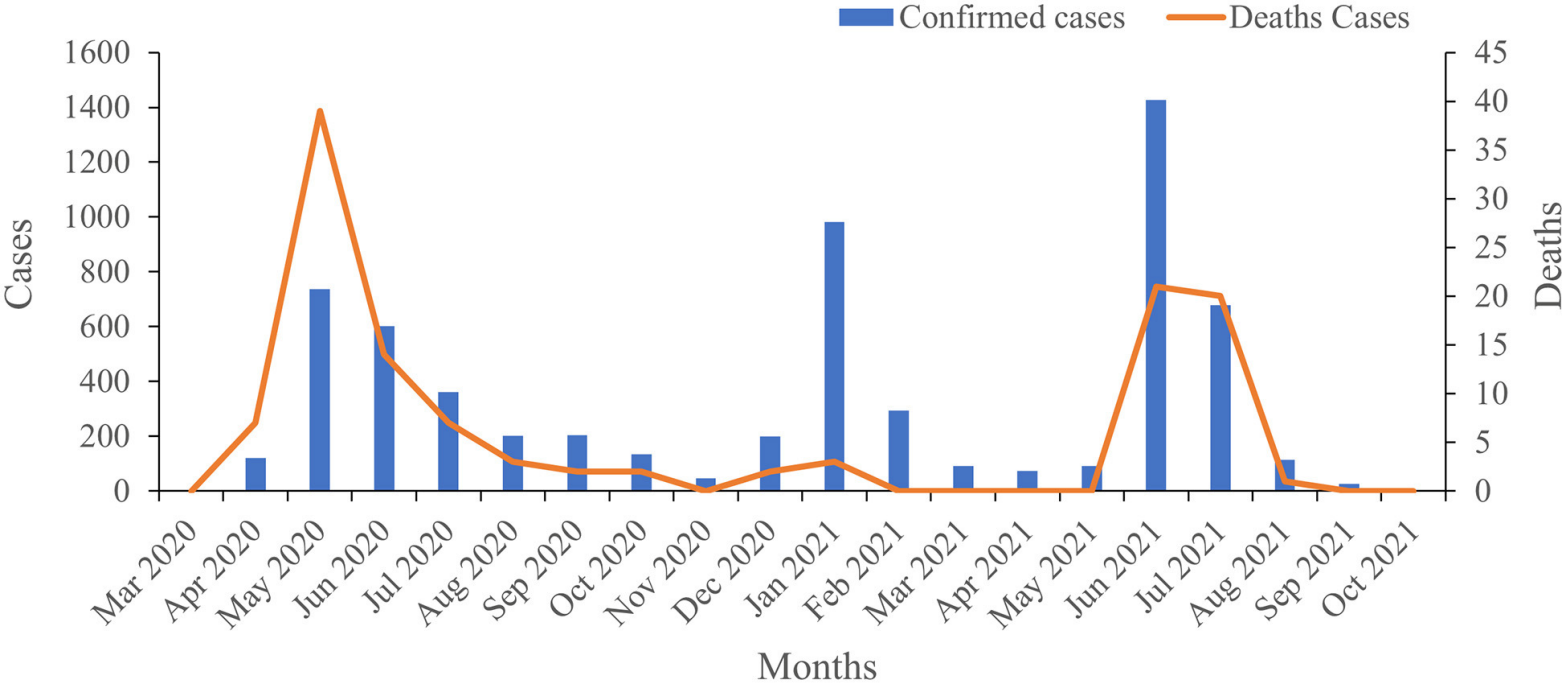
The changing trend among confirmed case numbers and deaths.

A total of 249,534 suspected cases of COVID-19 were tested during the survey period, and 6,398 samples were positive for SARS-CoV-2 infection, for a positivity rate of 2.56% (Figure 2). In 2020, the highest positivity rate was observed in May 2020 (16.89%), followed by in June (11.28%), April (6.57%), and July (6.10%), respectively. In 2021, the highest positivity rate was observed in June (7.36%), followed by in January (5.04%) and July (3.32%), respectively. There was no increase in the number of confirmed COVID-19 cases concurrent with increasing testing of samples, except during the third wave. Moreover, deaths were obviously related to the number of confirmed cases in this study; all of the recorded deaths happened during the three waves of COVID-19, with 74 deaths occurring in the first wave, five in the second wave, and 42 in the third wave, respectively (Figure 3).

### Geographic Distribution Features of Both Confirmed Cases of COVID-19 and Related Deaths

Confirmed cases were observed in all 16 regions of Sierra Leone, with 57.90% (3,705/6,398) of cases distributed in the urban western area and 16% (1,026/6,398) of cases found in the rural western area (Table 1). The case positivity rates in Kenema, Port Loko, and Bo were 2.8% (176/6,398), 2.5% (163/6,398), and 2.3% (148/6,398), respectively. The remaining cases were distributed in the other 11 regions. Moreover, the number of positive cases found among incoming travelers was 409 (4.6%) (Table 1). Deaths were reported in eight regions, with 82.6% (100/121) of deaths recorded in the urban western area and six deaths recorded in the rural western area. The remaining 15 deaths were distributed in six different regions, including Kenema (*n* = 4), Bombali (*n* = 4), Port Loko (*n* = 2), Bo (*n* = 2), Kono (*n* = 2), and Moyamba (*n* = 1).

**Table 1:** Geographic distribution features of both confirmed COVID-19 cases and deaths.

**Districts**	**CCC**	**PTC**	**CD**	**Cases/100,000 people**
Urban western area	3,705	57.90%	100	308.7
Rural western area	1,026	16.00%	6	201.5
Port Loko	163	2.50%	2	26.3
Kenema	176	2.80%	4	26.2
Tonkolili	111	1.70%	0	19.4
Bombali	81	1.30%	4	16.8
Bonthe	122	1.90%	0	54.4
Bo	148	2.30%	2	40.5
Koinadugu	51	0.80%	0	18.5
Falaba	11	0.20%	0	5.7
Kailahun	113	1.80%	0	18.3
Moyamba	77	1.20%	1	21.1
Pujehun	51	0.80%	0	12.8
Kono	106	1.75%	2	18.7
Kambia	37	0.60%	0	9.4
Karene	11	0.20%	0	5.2
Incoming passengers	409	6.40%	0	-
Total	6,398	-	-	83.5

*CCC, cumulative confirmed cases; CD, cumulative deaths; PTC, proportion of total cases; “-”, no data*.

### Relationship Between Age, Gender, and Case Fatality Rate

**Figure 4:**
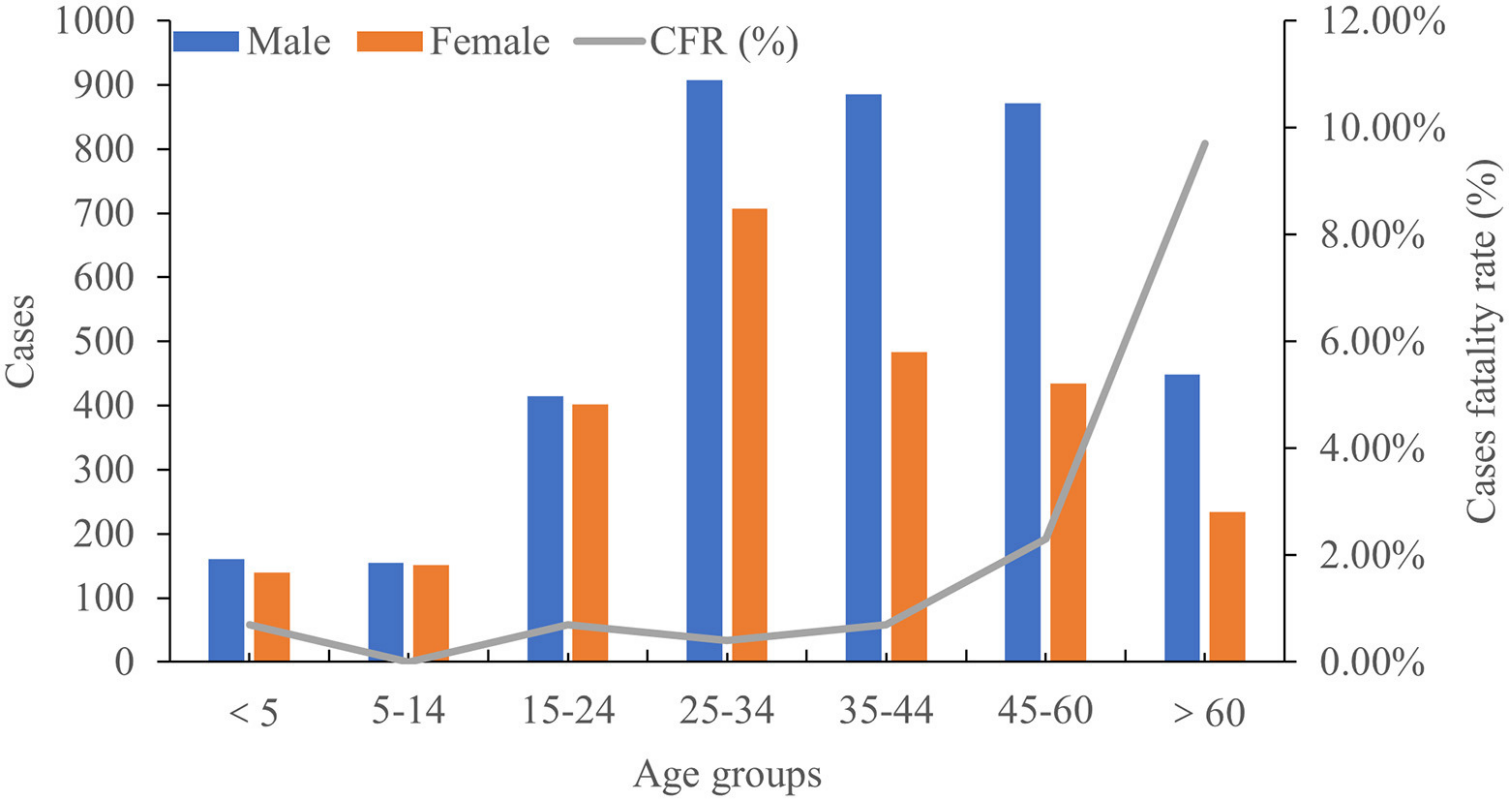
Distribution profile of age groups and case fatality rate.

Among 6,398 cases, 60% of patients were male and 40% of patients were female (Figure 4). The median age among all cases was 35 years (range, 1–98 years), with median ages of 33 years among female patients and 37 years among male patients. Approximately 73% of patients were <45 years of age. During our study period, 60% of deaths occurred among men, and the median age of patients who died was 64 years (Figure 4). The overall CFR in this study was 1.9%, and CFR in both female and male all were 1.9%, with no statistical difference according to gender observed. However, the CFR of patients aged >60 years was 9.7%, and the CFR among those 45–60 years of age was 2.3% (Figure 4).

### Epidemiological Analysis of Numbers of Incoming Travelers and the Epidemic Situation

**Figure 5:**
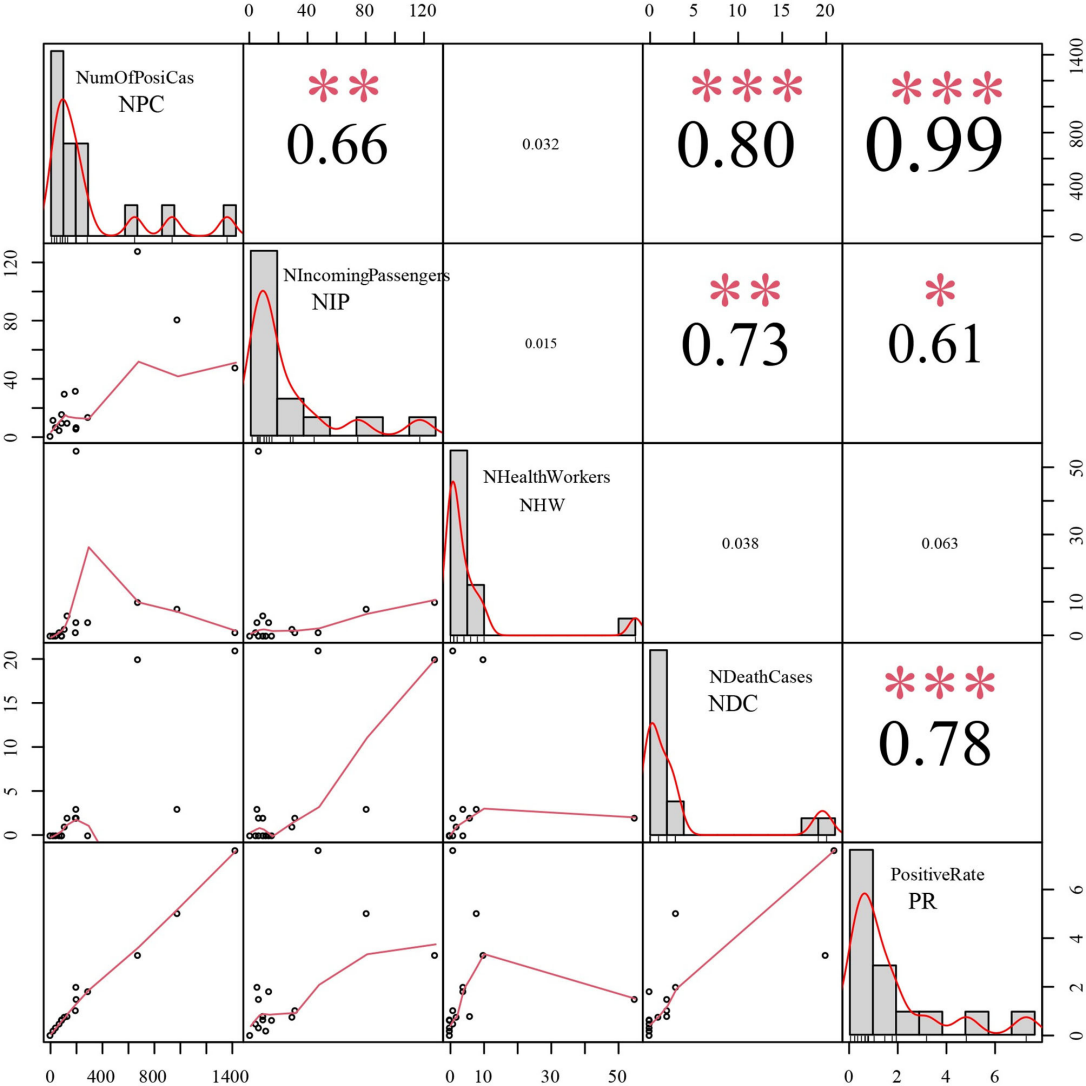
The epidemiological correlation analysis between positive cases among incoming travelers and the other 4 epidemic indices. NPC: Number of positive cases, NIP: Number of positive cases among incoming passengers, NHW: Number of positive cases in health workers, NDC: Number of death cases, PR: Positive rate of tested samples, CC: correlation coefficient. *** refer to *P* < 0.001; ** refer to *P* < 0.01; * refer to *P* < 0.05.

The first imported case was reported on March 31, 2020. Then, there were no positive cases among incoming travelers identified from March to July 2020; however, a total of 409 positive cases among incoming travelers were recorded from August 2020 to October 2021. Our epidemiological analysis revealed that the number of positive cases had a significant correlation with positive case numbers among incoming travelers [correlation coefficient (CC) = 0.66, *P* < 0.01], deaths (CC = 0.80, *P* < 0.01), and the positivity rate of tested samples (CC = 0.99, *P* < 0.01) (Figure 5). Moreover, the positive case numbers of incoming travelers showed a significant correlation with numbers of deaths (CC = 0.73, *P* < 0.01) and the positivity rate of tested samples (CC = 0.61, *P* < 0.01) (Figure 5). Meanwhile, there was a significant relationship between the number of deaths and the positivity rate of the tested samples (CC = 0.78, *P* < 0.01) (Figure 5).

## Discussion

In the present study, a comprehensive epidemiological feature analysis of COVID-19 cases in the setting of a medical resource–scarce country (Sierra Leone) was performed. From the start of the pandemic in 2020 until October 2021, a total of 249,534 samples of suspected COVID-19 cases were tested, and the average positivity rate of all samples was 2.56%. Both the number of total tested samples and the positivity rate was significantly less than those in other African countries, suggesting that the COVID-19 might be not widely spreading, revealing that relatively successful curbed spread of COVID-19 in Sierra Leone. This finding was consistent with the report from Barrie et al. (16), the overall weighted seroprevalence was 2.6% in Sierra Leone in March 2021, and overall seroprevalence was low compared with countries in Europe and the America. Similarly, results from a previous report showed that there were relatively lower incidence rates across three countries in Africa (Sierra Leone, Guinea, and Liberia) compared to many parts of the globe (17). In Nigeria, there were 802,143 tests conducted from February 2020 to April 2021, with a resulting positivity rate of 3.2%, and 66,121 and 91,644 people tested positive for SARS-CoV-2 infection during two waves (18). As of December 31, 2020, southern regions in Africa began reporting more cases (43%) than its eastern and northern regions; in contrast, fewer cases were being reported in western African (9%) (19). The effects of the COVID-19 pandemic in Africa have not been very devastating thus far, which may be connected to the early response strategies. The first COVID-19 case in Sierra Leone was recorded on March 31, 2020, in an individual with a history of travel to France before the onset of illness. First, in order to prevent imported cases of COVID-19, some targeted control measures, including closed borders, restricted (or suspended) international air traffic, or imposed travel restrictions to and from specific countries, were launched (20). Subsequently, national lockdowns took place; social distancing, mandatory mask-wearing policies, and hand-hygiene strategies were massively disseminated; public gatherings were banned; and public spaces were closed. Further, from April 14 to July 4, 2020, there was a ban on inter-district travel, and traveler quarantine requirements were enacted. West Africa in particular had learned lessons from its experience with the 2014 Ebola virus disease epidemic (21). To some extent, these prevention measures helped to effectively mitigate the spread of SARS-CoV-2. Previously reported data also show that malaria-endemic regions had a low prevalence of COVID-19 due to the fact that malaria might trigger protection against SARS-CoV-2 infection or contribute to better outcomes of the disease (22, 23). This may partly explain why Sierra Leone did not experience similar infection or fatality rates as those recorded in other parts of the world.

Despite this, there were still three waves of serious COVID-19 case numbers recorded from 2020 to October 2021. Especially, the first wave of the pandemic remained ongoing for up to six months, and it thoroughly changed the transmission pattern of the disease from sporadically imported cases to domestic clusters and community transmission (24). Therefore, the detected positive rate of COVID-19 in the pandemic's early stage is higher than later, which reveals that the COVID-19 epidemic is more severe in the early stage. However, some false-positive and false-negative results are observed in the COVID-19 test by RT-PCR (25, 26), so some misleading results cannot be excluded in our detection. Because SARS-CoV-2 detection assays would differ in terms of sensitivity, specificity and/or accuracy, so we recommend implementing future epidemiological studies based on a combination of diagnostics tests (27).

Although there was a relatively short period of disease spread in the second and third waves, >70 confirmed cases were reported every week on average. The COVID-19 epidemic trend in the regions analyzed herein was the same as that in other African countries at the peak of the first wave in Africa in July 2020. As of December 31, 2020, 40 (73%) countries in Africa had experienced or were experiencing their second wave of cases (19). Moreover, after April 2021, some countries in Africa experienced a surge in the number of COVID-19 cases and related deaths in what is now referred to as the continent's third wave of the pandemic (28). Furthermore, the three waves of COVID-19 in Sierra Leone occurred later on than in other African countries, and 6.4% (409/6,398) of cases arose from incoming travelers. Interestingly, the positive numbers of incoming passengers significantly correlated with the numbers of deaths and the positivity rate of tested samples. It is important to note that there was no increase in the numbers of confirmed COVID-19 cases alongside increased testing, except during the third waves. Moreover, the spikes in the incidence and mortality rates during the third wave of COVID-19 in Africa were not associated with an increase in diagnostic COVID-19 testing (28). This suggests that SARS-CoV-2's introduction led to local transmission events. A previous study showed that, in Sierra Leone, the second COVID-19 wave was mainly caused by the R1 lineage, while the third COVID-19 wave was dominated by the spread of the B.1.617.2 lineage (Delta variant), and further phylogenetic analysis revealed that multiple introductions of SARS-CoV-2 into Sierra Leone subsequently led to clusters and community transmission in the country (29). Similarly, most of Africa's COVID-19 cases that were considered to constitute the third wave of the pandemic were either imported cases or were triggered by imported COVID-19 cases (28). Moreover, based on the data from “Our World in Data,” around 1,284 new cases were reported from Nov 2021 to May 2022 in Sierra Leone. We consider this as the fourth wave of infection caused by Omicron and (or) other variants (30). Therefore, we recommend testing the virus strain in parallel with the epidemiological monitoring of the waves, as it is definite that the virus variants could impact the epidemiological trend. Moreover, the CFR was 1.9%, and all of the deaths occurred during each peak in the three waves of COVID-19. The low in CFR was associated with active engagement in the national preparedness response plan to ensure a resilient referral system, which made responders able to effectively manage the sudden demands of the referral of COVID-19–related cases (7). The relatively lower toll of COVID-19 in Africa may be due to differences in genetic or climatic factors or other potential driving factors, such as under-reporting (31, 32).

The various COVID-19 waves were exacerbated by different human behaviors, inertia governmental actions and rules, travel, daily activities, and the emergence of novel variants (28). In order to prevent SARS-CoV-2 from further spreading and expanding, there is a need, therefore, to intensify ongoing efforts, except for some of the existing measures. First, masks alone will not eliminate COVID-19 and they are not a substitute for vaccines. However, when COVID-19 mortality rates are high and health systems are strained, masks can potentially save many lives at a low cost (33). Second, although lockdown measures may have helped to inhibit community transmission, they also harmed health by affecting the functioning of the health system and causing social and economic disruption (34). Hence, this measure should be contextually tailored, taking local behavioral norms into consideration, and be community-led (35). Physical distancing can mitigate SARS-CoV-2 transmission (36) and can actually reduce the reproduction number of SARS-CoV-2 to <1 (37), but achieving this measure in overcrowded communities can be very challenging. Indeed, the highest R0 value was observed before every wave (range, 1.23–1.91) in our study. One in three Africans is living below the global poverty line (38). Most deprived communities lack running water, toilet facilities, soap, and basic food items (39). Thus, it may be a difficult chore for Africans to follow COVID-19 precautions, such as physical distancing, hand hygiene, and the wearing of face masks (28). Therefore, we suggest that the vaccination rate should be increased by improving the vaccine supply, population awareness about infection with the new viral variants and its complications, particularly the long-term COVID-19.

The proportion of children among COVID-19 infected cases seen in a rural community in Sierra Leone was 30%, and malaria was confirmed in 40% of the infected children (40). We suggest that medical resources be allocated among children and older individuals, and the interactive impact of malaria, human immunodeficiency virus/tuberculosis, and COVID-19 should be further investigated (31). This will enable us to better manage those who present with fever during the COVID-19 pandemic. Finally, continuous strengthening of incoming traveler testing and implementing a strict positive traveler case-management system will help to contain the spread of SARS-CoV-2 at the source of origin.

This study has several limitations. First, the number of reported confirmed cases enrolled in this study may be lower than the actual number of infections due to restraints in testing capacity and the number of tests available in the country. Second, the majority of confirmed cases and deaths being from the urban western area may only partly reflect the true epidemiological trends of the country, and further investigation in other regions is essential. Finally, clinical manifestations in confirmed and deaths were not considered in the present study, so further survey of these factors could help enhance timely case management and treatment.

## Conclusion

This study performed an epidemiological trend analysis of COVID-19 in the medical resource–scarce country Sierra Leone. Although the COVID-19 caseload in Sierra Leone remained on the lower end, a lack of sufficient data, asymptomatic infections, an imbalance in data availability between different regions, and poor reporting practices in the country limited our comprehensive understanding of COVID-19's trends. However, analysis of these data over time will provide valuable clues in an attempt to balance control of the transmission of COVID-19 with ensuring a stable socio-economics order.

## Data Availability Statement

The original contributions presented in the study are included in the article/supplementary material, further inquiries can be directed to the corresponding authors.

## Author Contributions

ZLiu and LG performed the data collection, process, and drafted the manuscript. ZLiu, DH, and ZLi participated in the design of the study. CX, CZ, and TL performed the critically reviewed the manuscript. JS, LW, DH, and AT participated in the design of the study and also managed the project. All authors read and approved the final manuscript.

## Funding

This study was supported by the National Key R&D Program of China (Grant Numbers: 2019YFC1200700, 2019YFC1200601-6, and 2021YFC2401000), the Third Phase of Technical Assistance Project for the Fixed Biosafety Laboratory in Sierra Leone from China CDC, and the National Natural Science Foundation of China (No. 82073624). The Youth Science Foundation of the State Key Laboratory of Infectious Disease Prevention and Control (Grant Number: 2021SKLID503). The funders had no role in study design, data collection and analysis, decision to publish, or preparation of the manuscript.

## Conflict of Interest

The authors declare that the research was conducted in the absence of any commercial or financial relationships that could be construed as a potential conflict of interest.

## Publisher's Note

All claims expressed in this article are solely those of the authors and do not necessarily represent those of their affiliated organizations, or those of the publisher, the editors and the reviewers. Any product that may be evaluated in this article, or claim that may be made by its manufacturer, is not guaranteed or endorsed by the publisher.
